# The Effects of Chromium and Vanadium on Biomarkers of Carbohydrate and Lipid Metabolism in Workers Exposed to Coal Fly Ash

**DOI:** 10.3390/jox12040021

**Published:** 2022-10-19

**Authors:** Lulzim Zeneli, Majlinda Daci-Ajvazi, Ankica Sekovanić, Jasna Jurasović, Demush Bajraktari

**Affiliations:** 1 Faculty of Education, University Fehmi Agani, 50000 Gjakova, Kosovo; 2 Faculty of Mathematics and Natural Sciences, University of Prishtina, 10000 Prishtina, Kosovo; 3 Institute for Medical Research and Occupational Health, 10000 Zagreb, Croatia; 4 Faculty of Pharmacy, UBT Higher Education Institution, 10000 Prishtina, Kosovo

**Keywords:** chromium, vanadium, carbohydrate, lipid, coal fly ash, correlation

## Abstract

Chromium (Cr) and vanadium (V) are micronutrients playing a role in carbohydrate and lipid metabolism but can be toxic at high concentrations, especially in specific forms. The study documents the effect of Cr and V concentrations on glucose and lipid metabolism in workers exposed to coal fly ash. We quantified selected metals (Cr, V) in the blood and serum of workers from a thermal power plant in Kosovo and compared them with the reference biological values. We determined fasting serum glucose and lipid profiles using a biochemical analyzer Synchron CX7 (Beckman Coulter). We quantified blood and serum Cr and V by inductively coupled plasma mass spectrometry. We also evaluated the association between carbohydrate and lipid metabolism biomarkers (glucose, cholesterol, and triglycerides) and co-exposure to coal fly ash. Power plant workers had significantly higher blood Cr and V levels (*p* < 0.0001) and significantly lower serum Cr and V levels (*p* < 0.0001) than the controls. We also found statistically significant (*p* < 0.0001) correlations between high blood Cr levels and low glucose/blood Cr ratios as well as between high serum Cr levels and low glucose/serum Cr ratios. Finally, in power plant workers, high blood V levels significantly correlated with low triglycerides/blood V and cholesterol/blood V ratios (*p* < 0.0001), while high serum V levels correlated with low cholesterol/serum V ratios (*p* = 0.005). Based on these findings, we concluded that the glucose/Cr, triglycerides/V and cholesterol/V ratios should be considered when evaluating carbohydrate and lipid metabolism disorders in occupationally-exposed workers.

## 1. Introduction

Human biomonitoring has its roots in the analysis of biological samples and can considerably help reduce uncertainty in health risk assessment [1,2,3]. Biological monitoring is defined as the repeated, controlled measurement of chemical or biochemical markers in biological samples or other accessible samples from subjects exposed to chemical, physical, or biological risk factors in the workplace and/or the general environment [4,5,6]. Cr and V are next to each other on the periodic table and share many characteristics [7,8]. Both of these elements, naturally present in our environment and predominantly excreted by the kidneys, are associated with normal human health and the pathogenesis of several diseases [9,10]. 

Cr and V metals do not occur naturally; instead, these elements are found in different valence states (Cr from +1 to +6 and V from −1 to +5) [11,12,13]. As transition metals, their chemistry is complex. They can participate in redox processes and produce free radicals [14], thus inducing lipid peroxidation both in vitro [15] and in vivo [16,17] and disturbing the antioxidative balance in the organism. Humans can be exposed to Cr and V through the air, but most of the exposure stems from food and water [11,12]. The hexavalent chromium form (Cr^6+^) easily enters cells through facilitated uptake, which is more efficient than the simple diffusion undergone by the trivalent form (Cr^3+^). It travels to and accumulates in the erythrocytes, but the highest Cr concentrations are found in the kidneys and liver [18,19]. Vanadium (V^5+^) readily enters cells via phosphate and sulfate ion channels; it also interferes with phosphate-containing enzymes [20], can activate several genes, and participates in the inflammatory response. The initial V concentrations are found in the kidneys, liver, and lungs, and in the long-term, it is stored in the bones and muscles [21]. The major route of elimination of absorbed Cr and V is urine, whereas unabsorbed Cr and V are excreted through the feces [22]. Vanadium has many systemic effects including gastrointestinal, respiratory, hematological, immunological, and cardiovascular effects, whereas trivalent and hexavalent Cr cause gastrointestinal, immunological, hematological (including anemia), reproductive, developmental, and other serious effects [12,23,24]. Vanadium compounds may prevent chemical carcinogenesis and act as potential anti-metastatic agents, and the estrogenic role of V and its possible therapeutic application in osteoporosis have also been described [25,26,27]. Supplementation with Cr decreases the plasma thiobarbituric acid reactive substance levels and minimizes the oxidative stress increase in patients with type 2 diabetes mellitus [28,29,30,31]. 

Several studies have shown that chromium and vanadium as transition metals have been directly implicated in the regulation of glucose and lipid metabolism, whereas increasing or decreasing levels of lipids and glucoses cause various health effects in the human body, which are called disorders. This study documents the effects of chronic exposure to anthropogenic pollution on the Cr and V levels and the relationship with lipid and carbohydrate metabolism biomarkers (glucose (Glu), cholesterol (Chol), and triglycerides (TG)) in the blood of power plant workers.

## 2. Materials and Methods

### 2.1. Study Population 

The main characteristics of the study population have previously been reported [32]. Blood samples from 100 male workers participating in the study were available for metal analysis. The age of the subjects ranged from 30 to 65 years (mean 51 years), while the occupational exposure duration ranged from 4 to 43 years (mean 21 years). Among the 100 subjects, 70 were male workers employed at the thermal power plant (ThPP) in ‘‘Kosova’’ (Obiliq, Kosovo) who had been exposed to traces of heavy metals, and 30 were healthy inhabitants from the rural municipality of Dragash, Kosovo (environment without pollutants found at the power plant). The study protocol was carried out in accordance with the regulations in force of the University Fehmi Agani, Gjakova, Kosovo, and prior written consent was obtained from each participant in the research. Each subject completed a questionnaire regarding age, occupational exposure duration, and medical history. The candidates declared that they did not have any known acute or chronic diseases and in addition, each subject was informed about the purposes of the study. The exclusion criteria from the study were as follows: alcoholism, gender, food, and lifestyle of the study group.

### 2.2. Blood Sampling and Trace Elements Analysis 

We sampled the venous blood of all subjects between 8:00 am and 9:00 am. Next, we placed 15 mL of blood in a K_2_EDTA-containing tube (BD Vacutainer K_3_E) to quantify the blood Cr (BCr) and blood V (BV) levels. We also placed the same amount of blood in an anticoagulant-free tube (BD Vacutainer Trace Element Serum, Heidelberg, Germany) to quantify the serum Cr (SCr) and serum V (SV) levels and into an anticoagulant-free tube (BD Vacutainer, SARSTEDT—Nümbrecht, Germany) for the biochemical parameter analysis. After allowing for 60 min of spontaneous blood clotting, we separated the serum from the blood cells via centrifugation at 3000 rpm/h for 10 min, decanted again, and stored in a metal-free polypropylene tube at −20 °C until SCr and SV quantification. We took special care to avoid any contamination with metals during the blood sampling, storage, and analyses. All chemicals used were of analytical grade for spectroscopy (Merck, Darmstadt, Germany). We quantified trace elements using inductively coupled plasma mass spectrometry [33]. We used a biochemical analyzer Synchron CX7 (Beckman Coulter Company, Indianapolis, IN, USA), with reagents obtained from Beckman Instrumental, Inc. (Galway, Ireland) to determine the biochemical parameters such as fasting serum glucose and lipid profiles. 

### 2.3. Statistical Methods

Because of the skewed distribution of most of the measured parameters, we presented the results within groups as the median and range, while we calculated the difference between groups using the *t*-test (*t*, *p*). We employed Pearson’s correlation (r, *p*) to explore the relationships between each of the measured parameters. The hypothesis testing in the statistical analysis was based on a 0.05 significance level. 

## 3. Results

The blood concentrations of metals and biological markers in the individuals studied in the present work are summarized in Table 1. 

Workers exposed to coal fly ash had significantly higher BCr and BV levels (*p* < 0.0001) and significantly lower SCr and SV levels (*p* < 0.0001) than the control subjects. The BCr and BV concentrations were 1.4 and 1.5 times higher in the study group than in the control group, respectively. Moreover, the SCr and SV concentrations were both 1.2 times lower in the study group than in the control group. Table 2 presents the biological markers to trace elements ratios. 

The power plant workers had significantly lower Glu/BCr, Chol/BCr, TG/BCr, Glu/BV, Chol/BV, and TG/BV ratios (*p* ≤ 0.0001) and significantly higher Glu/SCr and Glu/SV ratios (*p* ≤ 0.005) than the control subjects. The Pearson’s correlation analysis (Table 3) showed that high BCr levels were significantly (*p* < 0.001) associated with low Glu/BCr ratios (r = −0.713). We observed a similar relationship with the SCr levels and the Glu/SCr (r = −0.678), Chol/SCr (r = −0.955), and TG/SCr (r = −0.358) ratios. Meanwhile, we found no significant correlation between the BCr levels and the Chol/BCr or TG/BCr ratios.

**Figure 1:**
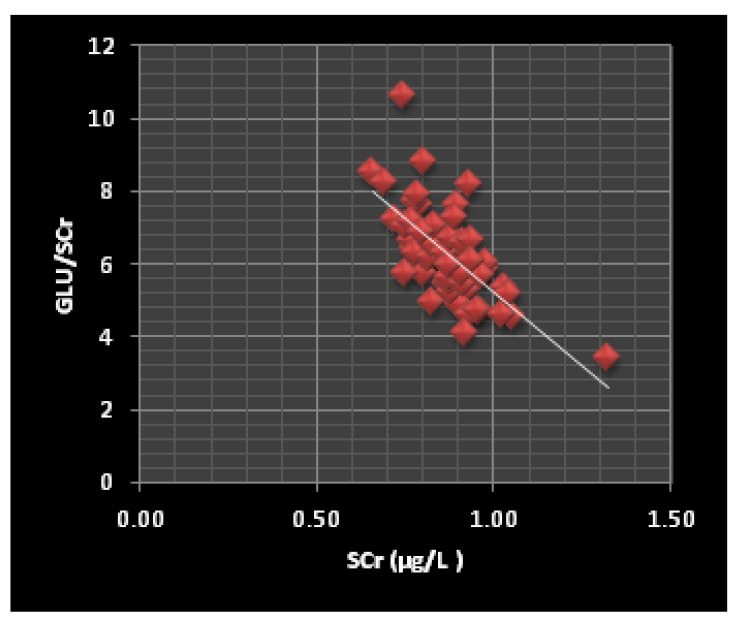
The correlation between the SCr levels and the Glu/SCr ratio in ThPP workers (r = −0.67, *p* < 0.0001).

Finally, in ThPP workers, high BV concentrations were significantly correlated (*p* < 0.05) with the low Glu/BV (r = −0.895), Chol/BV (r = −0.825), and TG/BV (r = −0.672) ratios. Similarly, high SV concentrations (Figure 1) were associated with the low Glu/SV (r = −0.288) and Chol/SV (r = −0.203) ratios. In contrast, we found no significant correlation between the SCr levels and TG/SV ratios.

## 4. Discussion

Cr and V are micronutrients that play a significant role in health maintenance [34], but excessive concentrations can lead to the development of diseases [35,36]. Vanadium pentoxide (V_2_O_5_), as a component of particles derived from the combustion of different types of fuel, and Cr^6+^, generated in the process of burning coal at high temperature in thermal power plants as the dominant form in fly ash, are a source of occupational exposure in humans [37,38,39,40,41]. In the present study, these forms of the two elements were analyzed, and we assessed the effect of V and Cr co-exposure on the carbohydrate and lipid metabolism biomarkers. Biomarkers are associated with an alteration in the cellular or biochemical components, processes, structures, or functions. Moreover, biological indicators can be any quantifiable substance, structure, or process tissues or fluids that reflect the health or the incidence or biological behavior of a disease [42]. For this purpose, we calculated the ratios of Glu, Chol, and TG to the Cr and V blood concentrations. Trace elements, especially Cr and V, play a significant role in the metabolism of lipids and carbohydrates [43,44]. Table 1 shows the relevant parameters of the investigation group. The investigation group had been significantly more exposed to nonessential elements than the control group, as indicated by the higher BCr and BV concentrations (*p* < 0.0001) and significantly lower SCr and SV concentration (*p* < 0.0001).

The higher BCr levels of the investigation group could be due to the propensity of erythrocytes to bind and uptake Cr(VI) [45]. Cr(VI) enters erythrocytes through a sulfate ion channel. Inside the cell, it is rapidly reduced into the reactive intermediates Cr(V) and Cr(IV) and binds to the beta chain of human hemoglobin [45,46,47] and other ligands such as proteins and glutathione. Furthermore, the lower SCr levels of the investigation group can be explained by the fact that ascorbate and glutathione can reduce inhaled Cr(VI) to Cr(III) in the epithelial lining fluid of the lungs [48,49]. The higher BV levels in the study group compared with the control group (Table 1) may result from vanadium emissions through coal fly ash. For most organisms including mammals, V is not known to have any essential biological function. However, many biomolecules contain vanadium. 

According to occupational exposure studies, human experimental studies, and laboratory animal studies, the respiratory tract is one of the primary targets of V toxicity after exposure by inhalation. Experimental studies indicate that, inside erythrocytes, the vanadium (V^5+^) is mainly bound to hemoglobin [50,51,52], but probably also to other intracellular bioligands [53,54]. The vanadium biomedical importance has been proven by numerous studies and is mainly based on its interaction with proteins, along with enzymatic systems and cellar components, which can affect the synthesis of lipids and lipoproteins or interact with catabolism [55].

In contrast, the lower SV levels observed in the study group (Table 1) compared with the control group may result from an interconversion between vanadium species (mostly V^4+^/V^5+^ and to a lesser degree V^+3^) inside the biomolecules [56,57,58]. 

We also evaluated the classification (ThPP workers vs. control group) performance of the Glu, Chol, TR, V, and Cr concentrations and ratios and performed a discriminant analysis (Table 1). The Glu/BCr, Chol/BCr, TG/BCr, Glu/BV, Chol/BV, and TG/BV ratios had smaller *p*-values (*p* ≤ 0.0001) than Glu, Chol, TR, V, or Cr concentrations, indicating that the biological markers to trace elements ratio had superior discriminating power over the Glu, Chol, TR, V, or Cr concentrations alone. The fact that the study group had significantly lower Glu/BCr, Chol/BCr, TG/BCr, Glu/BV, Chol/BV, and TG/BV ratios than the control group could indicate a higher risk of hyperglycemia and lipid metabolism disorders. 

Recent studies have demonstrated that Cr metabolism is affected by several factors including lifestyle (stress, diet, activities) and diabetes [36,59,60]. Additionally, chromium is well-known to play substantial roles in the lipid and metabolism of carbohydrates and affects the etiology of diabetes, obesity, and cardiovascular diseases [61]. 

**Figure 2:**
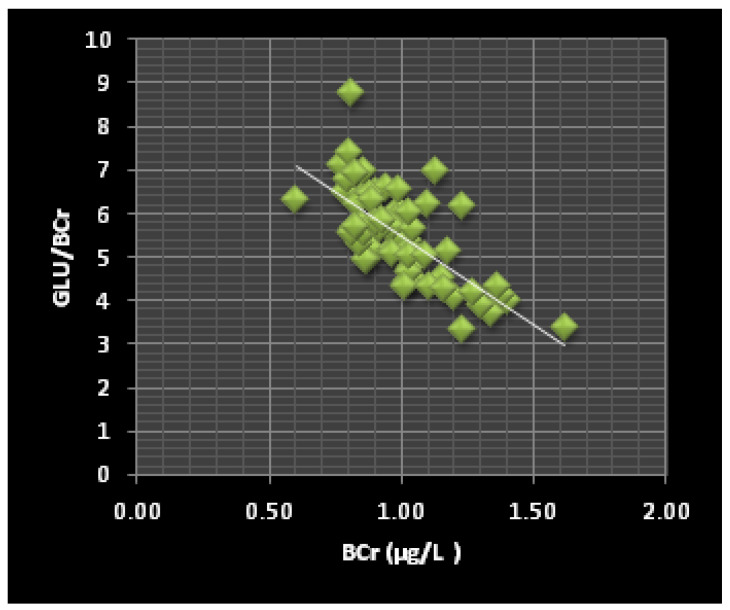
The correlation between the BCr levels and the Glu/BCr ratio in the ThPP workers (r = −0.70, *p* < 0.0001).

Our results show that a highly significant correlation exists between the BCr levels and Glu/BCr ratios (r = −0.713) as well as between the SCr levels and the Glu/SCr (r = −0.678), Chol/SCr (r = −0.955), and TG/SCr (r = −0.358) ratios. A correlation such as that between BCr and Glu/BCr (Figure 2) and SCr and Glu/SCr (Figure 1) could result from predispositions to diabetes, as the ability to convert inorganic chromium into a useable organic form (organic chromium) that activates insulin is reduced in people predisposed to diabetes [62,63,64]. Moreover, the study group displayed lower SCr levels than the control group (Table 1), which supports this hypothesis. In addition, the correlation between the SCr levels and the Chol/SCr and TG/SCr ratios may result from Cr metabolism and thus be a predictor of cardiovascular risk.

**Figure 3:**
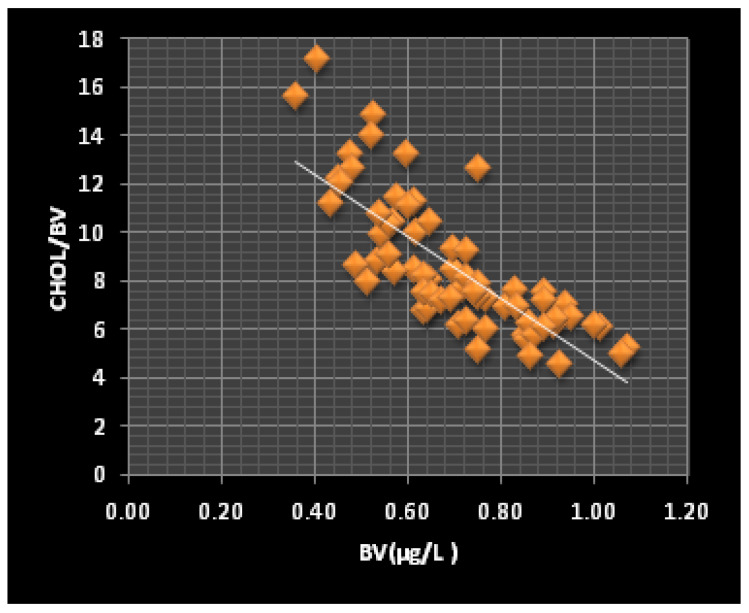
The correlation between the BV levels and the Chol/BV ratio (r = −0.78, *p* < 0.0001) in the ThPP workers.

**Figure 4:**
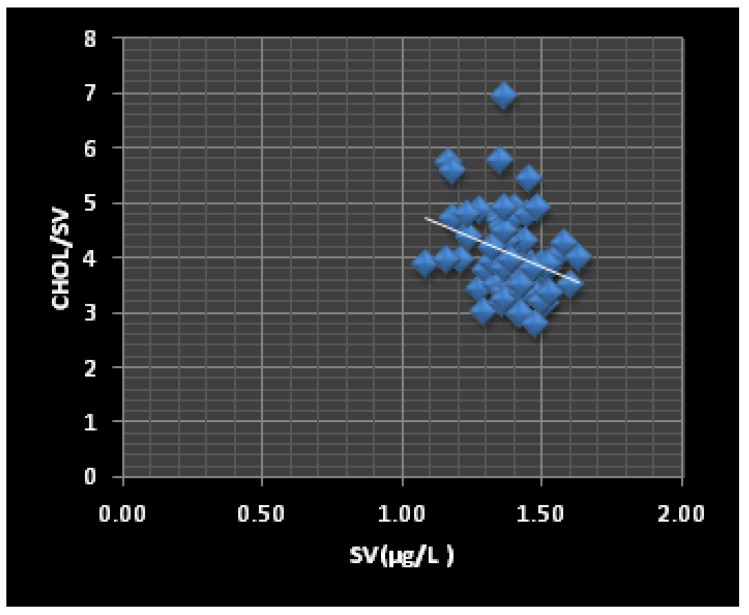
The correlation between the SV levels and the Chol/SV ratio (r = −0.31, *p* = 0.0048) in the ThPP workers.

In the investigation group, the BV concentration was significantly correlated (*p* < 0.05) with the Glu/BV (r = −0.895), Chol/BV (r = −0.825), and TG/BV (r = −0.672) ratios, while the SV concentration (Figure 1) was correlated with the Glu/SV (r = −0.288) and Chol/SV (r = −0.203) ratios. The role of V in carbohydrate hemostasis can explain the correlation between BV and Glu/BV and SV and Glu/SV. Recent studies have shown that administering V to diabetic animals or humans decreased hepatic glucose production [65,66,67]. Additionally, the direct biochemical control of glucose homeostasis during the V treatments is associated with the enhancement of glycolysis and glucose oxidation [68,69]. In contrast, the correlations between the BV and SV concentrations and the Chol/BV and Chol/SV (Figure 3 and Figure 4) and TG/BV ratios could be explained by the role of V in the process of reducing the total and free cholesterol levels, or through the inhibition of the cholesterol biosynthesis steps [70].

Glu/Cr and Tr/V and Chol/V ratios would be more valuable than the biochemical parameters (Glu, TR, Chol) levels alone for predicting the early stage of disorders of carbohydrate and lipid metabolism risk. Future studies should explore the underlying mechanisms of this relationship, compare genders (male and female), assess the impacts of alcohol consumption, and monitor the long-term impacts of exposure.

## 5. Conclusions

Our results indicate that the Glu/Cr, TG/V, and Chol/V ratios should be considered when evaluating carbohydrate and lipid metabolism disorders in occupationally-exposed workers. Furthermore, this study demonstrated that coal fly ash significantly altered the Cr and V blood levels. Cr affects the biochemical parameters, leading to various diseases. Nevertheless, the biochemistry of chromium and vanadium requires more exploration. The Glu/Cr, TG/V, and Chol/V ratios should be considered in the evaluation of disorders of carbohydrate and lipid metabolism expressly in the environments that are affected by several factors including environmental pollutions, stress, diet, etc.

## Figures and Tables

**Table 1:** Characteristics of the study groups based on the exposure duration of and concentrations of Cr and V in the blood and serum samples and biological markers.

Characteristic	ThPP Workers	Control Subjects	T	*p*
Total Number of Individuals	70	30	-	-
Occupational Exposure Duration (years)	21 (4–43)	0	-	-
Age (years)	51 (31–64)	42 (30–65)	0.67	<0.5
BMI	27.4 ± 0.17	26.9 ± 3.8	4.5	<0.0001
BCr (µg/L)	1.0 ± 0.3	0.73 ± 0.12	7.3	<0.0001
BV (µg/L)	0.7 ± 0.16	0.47 ± 3.6	6.6	<0.0001
SCr (µg/L)	0.87 ± 0.1	1.07 ± 0.07	−9.3	<0.0001
SV (µg/L)	1.38 ± 0.1	1.70 ± 0.07	−14	<0.0001
Glu (mmol/L)	5.62 ± 1.5	5.88 ± 1.4	−1.0	0.283
TG (µg/L)	1.65 ± 0.4	1.84 ± 0.5	−3.2	<0.002
Chol (µg/L)	5.63 ± 1.1	6.4 ± 1.0	−1.9	<0.05

**Table 2:** A comparison of the biological markers glucose, cholesterol, and triglycerides to the trace elements ratios of the study group (ThPP workers, *n* = 70) and the control group (*n* = 30).

	ThPP Workers	Control Subjects	T	*p*
Glu/BCr	5.62	8.05	−6.8	<0.0001
Chol/BCr	5.63	8.76	−7.9	<0.0001
TG/BCr	1.56	2.52	−5.7	<0.0001
Glu/SCr	6.45	5.49	3.4	<0.005
Chol/SCr	6.47	5.98	1.64	0.115
TG/SCr	1.79	1.71	1.13	0.275
Glu/BV	8.08	13.36	−9.1	<0.0001
Chol/BV	8.04	13.61	−9.3	<0.0001
TG/BV	2.22	3.91	−5.6	<0.0001
Glu/SV	4.19	3.45	−3.8	0.0002
Chol/SV	4.08	3.76	−1.65	0.105
TG/SV	1.13	1.02	−1.06	0.226

**Table 3:** The Pearson’s correlation coefficients and significance levels (r, *p*) for the relationships between the Cr and V concentrations and biological markers (glucose, cholesterol and triglycerides) to trace elements ratios in whole blood and serum.

	B ^1^ Cr (µg/L)	S ^2^ Cr (µg/L)	BV (µg/L)	SV (µg/L)
**Glu/BCr**	**−0.713 ^a^**			
Chol/BCr				
TG/BCr				
Glu/SCr		−0.678 ^a^		
Chol/SCr		−0.955 ^a^		
TG/SCr		−0.358 ^c^		
Glu/BV			−0.895 ^a^	
Chol/BV			−0.825 ^a^	
TG/BV			−0.672 ^b^	
Glu/SV				−0.288 ^d^
Chol/SV				−0.203 ^d^
TG/SV				

^a^*p* ≤ 0.00001; ^b^ *p* ≤ 0.0001; ^c^ *p* ≤ 0.001; ^d^ *p* ≤ 0.05; ^1^ B—blood; ^2^ S—serum.

## Data Availability

Not applicable.
